# Case report: Sentinel lymph node mapping of endometrial carcinoma occurring in uterine didelphys

**DOI:** 10.1016/j.gore.2021.100769

**Published:** 2021-04-30

**Authors:** Dib Sassine, Sara Moufarrij, Anjelica Hodgson, Sarah Ehmann, Nadeem R. Abu-Rustum, Sarah Chiang, Elizabeth L. Jewell

**Affiliations:** a Gynecology Service, Department of Surgery, Memorial Sloan Kettering Cancer Center, New York, NY, USA; b Department of Pathology, Memorial Sloan Kettering Cancer Center, New York, NY, USA; c Joan & Sanford I. Weill Cornell Medical College of Cornell University, New York, NY, USA

**Keywords:** Endometrial carcinoma, Lymphadenectomy, Uterine didelphys, Sentinel lymph node mapping

## Abstract

• In a bleeding postmenopausal woman with didelphys uterus, endometrial biopsy should be taken from both uterine cavities.
• Sentinel lymph node mapping has not been previously described in the setting of endometrial cancer and uterine didelphys.
• Routine sentinel lymph node mapping was successfully performed in a patient with endometrial cancer and uterine didelphys.

## 1 Introduction

Anatomical uterine anomalies are the most common type of congenital malformation in the female genital tract (Gao et al., 2017). Although endometrial carcinoma is relatively common, the presence of endometrial carcinoma in patients with anatomical uterine anomalies has been documented only rarely in case reports and case series (Gao et al., 2017, Vanichtantikul et al., 2020).

Surgical management of endometrial carcinoma has evolved in recent years with the use of sentinel lymph node mapping, which has improved detection of metastases while decreasing the morbidity associated with lymph node resections (Raimond et al., 2014). In this report, we describe sentinel lymph node mapping in the case of a patient with uterine didelphys and endometrial carcinoma. To our knowledge, sentinel lymph node mapping in this context has not been previously described in the literature.

## 2 Case report

**Fig. 1:**
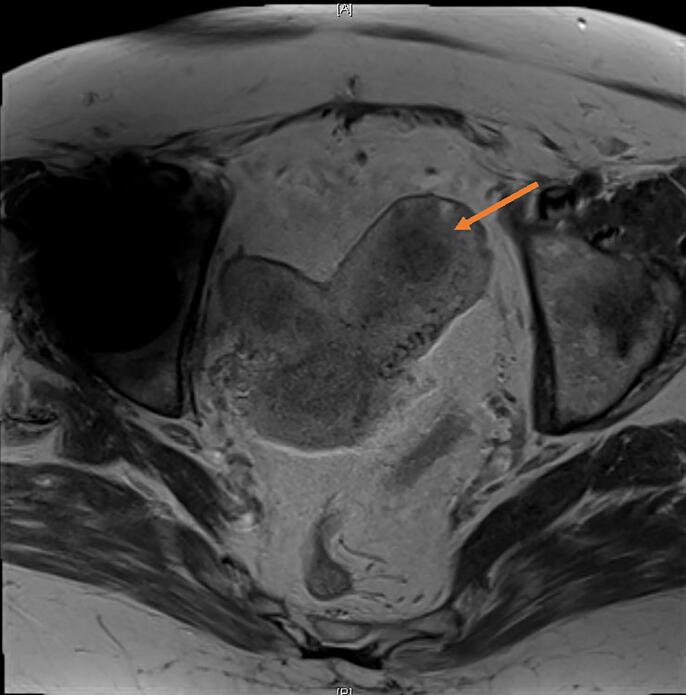
Magnetic resonance T1 axial image showing didelphic uterus with a mass in left uterine horn (orange arrow). (For interpretation of the references to colour in this figure legend, the reader is referred to the web version of this article.)

**Fig. 2:**
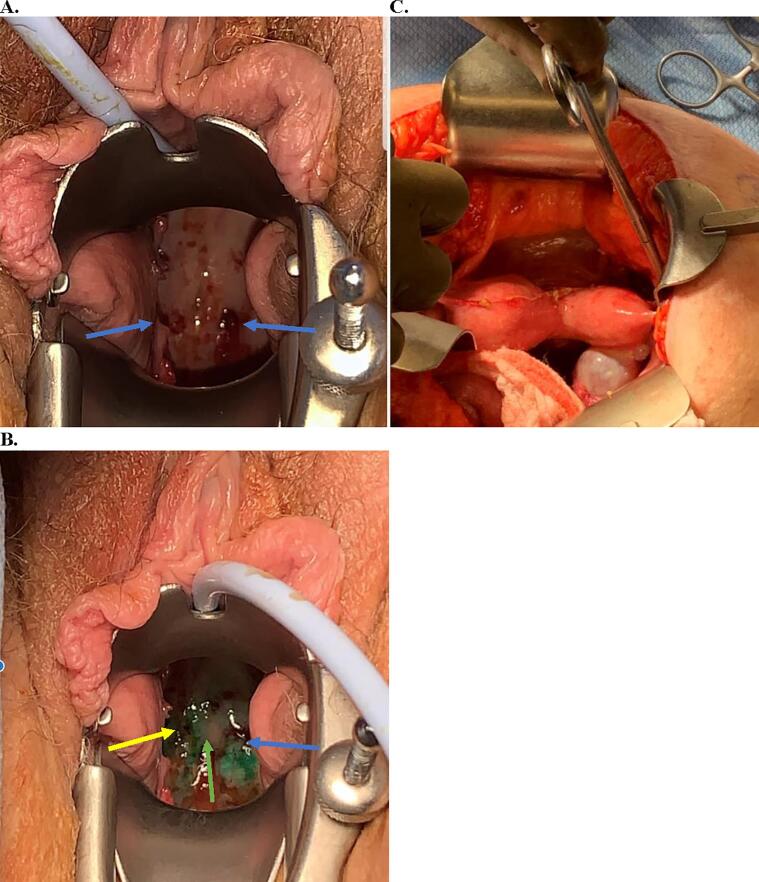
The two cervical osses can be seen (A, blue arrows). Injection of Indocyanine Green (ICG) dye at 1.25 mg/ml at 3o’clock of the left cervix (blue arrow) and 9o’clock of the right cervix (yellow arrow) and the midline fused portion (green arrow) of the cervices, with the dye seen around the circumference (B). Uterine didelphys shown with right adnexal cyst (C). (For interpretation of the references to colour in this figure legend, the reader is referred to the web version of this article.)

**Fig. 3:**
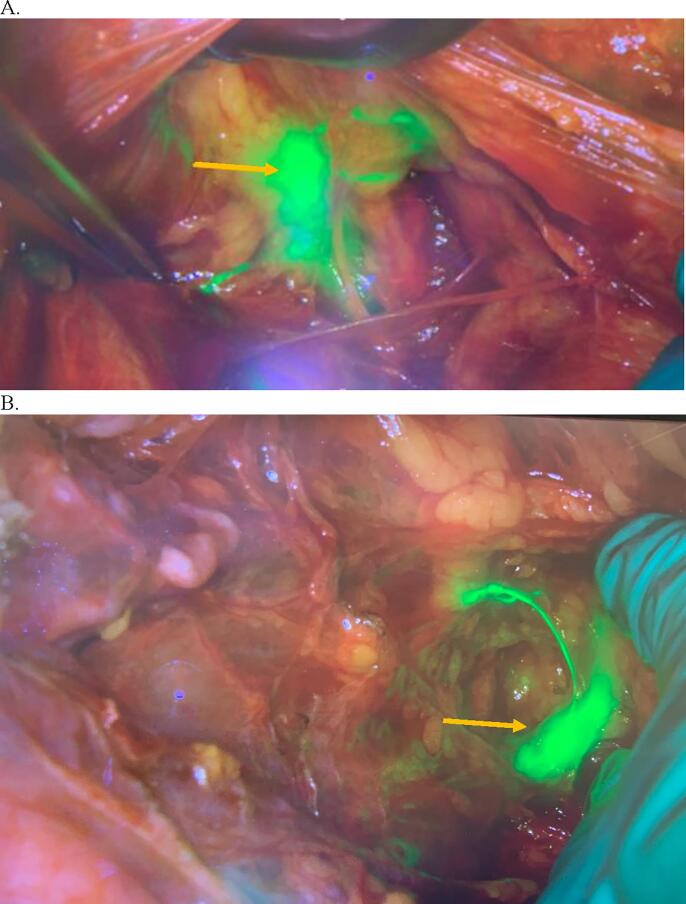
Sentinel lymph node mapping using Indocyanine Green (ICG) and near-infrared imaging. (A) Left-sided lymph node mapping with the left obturator sentinel lymph node (orange arrow) evident. (B) Right lymph node mapping with the right obturator sentinel lymph node as sentinel lymph node (orange arrow) with its channel in fluorescent green. (For interpretation of the references to colour in this figure legend, the reader is referred to the web version of this article.)

The patient, a 70-year-old woman, gravida 2 para 2, with a history of hypertension, overactive bladder, solitary left kidney and known uterine didelphys, presented for evaluation of postmenopausal bleeding. An ultrasound demonstrated the presence of a didelphic uterus with bilateral endometrial masses, as well as a 3.0 × 2.0 × 3.0 cm right adnexal cyst. Subsequent magnetic resonance imaging (MRI) showed an anteverted uterine didelphys with the left uterine horn measuring 8.1 × 3.3 × 3.9 cm and the right uterine horn measuring 7.8 × 3.0 × 3.2 cm. The left horn showed an endometrial thickness of 1.9 cm and a 3.2 × 2.1 × 2.4 cm mass, with deep myometrial invasion but no uterine serosal involvement (Fig. 1). The right horn showed an endometrial thickness of 1.5 cm, with a 1.6 × 1.1 cm polypoid filling defect found in the mid body. A simple ovarian cyst measuring 2.8 × 2.8 × 3.4 cm, involving the right ovary, was also seen. Office endometrial biopsies of the left and right uteri showed FIGO grade 1 endometrioid adenocarcinoma, and rare atypical cells with tissue fragments thought to be in keeping with an endometrial polyp, respectively. The patient underwent an exploratory laparotomy, total abdominal hysterectomy, bilateral salpingo-oophorectomy, omentectomy, pelvic washings, and sentinel lymph node biopsy, with the removal of bilateral obturator sentinel lymph nodes per the sentinel lymph node mapping algorithm (Barlin et al., 2012). Indocyanine green (ICG) was used for this purpose as per National Comprehensive Cancer Network (NCCN) guidelines. The patient’s cervical anatomy showed a right and left cervix that were fused medially. Unique to this procedure, injection sites were placed at the 3o’clock position of the left cervix, the 9o’clock position of the right cervix, and the mid fusion point of both cervices, as shown in Fig. 2. A total of 6 mL of ICG was used. Near-infrared detectors were used to map the lymphatic drainage (Fig. 3). Sentinel lymph nodes were visualized on both the right and left pelvic sidewalls and were sampled. The didelphic uterus, bilateral tubes and ovaries (Supplementary Fig. 1A), omentum, and bilateral sentinel lymph nodes were submitted for pathological examination.

The left uterus harbored a FIGO grade 1 endometrial endometrioid adenocarcinoma (Supplementary Fig. 1B). The depth of myometrial invasion was 11 of 22 mm. No cervical stromal, uterine serosal, or lymphovascular invasion was identified. The right uterus harbored a uterine adenomyoma. Other benign uterine findings included adenomyosis and leiomyomas. The right ovary harbored a benign simple cyst. The left ovary, bilateral fallopian tubes, and bilateral cervices were unremarkable. The sentinel lymph nodes (one node from the left obturator region and one node from the right obturator region) were pathologically ultrastaged as previously described (Kim et al., 2013) and found to be negative for metastatic carcinoma. Pelvic washings and the omentum were also benign.

The final stage was FIGO 2018 stage IB. The patient was discharged on post-operative day 3. On follow-up after 6 weeks she was found to be doing well and is receiving intravaginal brachytherapy.

## 3 Discussion

Uterine didelphys, a rare anatomical anomaly thought to occur in 1/3000 women (Grimbizis et al., 2001) (although its prevalence is likely underreported), is a duplication of the uterine horns and cervix. This occurs secondary to a lateral fusion defect, due to failure of the formation of one of the two Müllerian ducts, or else as a result of the failure of fusion of the Müllerian ducts at approximately 12 weeks’ gestation, ultimately resulting in a duplicated reproductive system. As Müllerian duct development is associated with development of the mesonephric duct, urinary tract anomalies such as unilateral renal agenesis can be found in about 30% of patients with uterine didelphys (Hall-Craggs et al., 2013). As noted, our patient had a solitary kidney.

A study by Gao et al in 2017 reviewed 25 patients with uterine anomalies and endometrial carcinoma and found that uterine didelphys was the most common anomaly in this group (Gao et al., 2017). However, endometrial carcinoma in uterine didelphys has been reported in only a few case reports and case series (Gao et al., 2017, Molpus et al., 2004). When endometrial carcinoma is present in a didelphic uterus, it usually occurs in only one of the uterine horns (Vanichtantikul et al., 2020, Molpus et al., 2004). For this reason, when performing preoperative biopsies to rule out neoplasia, both uterine horns should be sampled because a negative finding in one does not exclude the presence of a neoplastic process in the other.

Typically, endometrial carcinoma is surgically staged, and lymph node involvement is a major factor in determining the need for adjuvant treatment such as chemotherapy and/or radiotherapy. Unfortunately, lymphadenectomy may lead to increased postsurgical complications including lymphedema, nerve and vascular injury, and deep vein thrombosis. Some retrospective studies have shown that multi-site and more extensive lymph node sampling was a significant prognostic factor for improved survival in patients with endometrial carcinoma (Chan et al., 2006). Follow-up randomized trials, however, have demonstrated that lymphadenectomy confers no survival benefit (Kitchener et al., 2009).

In order to mitigate the morbidity associated with lymphadenectomy while still allowing assessment of nodal disease, a sentinel lymph node surgical algorithm was published in the 2014 NCCN guidelines for endometrial carcinoma. This focused on (1) peritoneal and serosal evaluation and washings, (2) retroperitoneal evaluation, including the removal of all sentinel lymph nodes and any other suspicious nodes, and (3) a side-specific pelvic, common iliac and interiliac lymph node dissection if there was no mapping in a hemipelvis. A *para*-aortic lymphadenectomy is done at the surgeon’s discretion. When routine hematoxylin and eosin (H&E) pathological evaluation is negative, ultrastaging is incorporated; this consists of cutting two consecutive 5 µm-thick sections from each paraffin block at two tissue levels 50 µm apart. At each level, one slide is stained with H&E and the other with AE1/AE3 by immunohistochemistry. With ultrastaging, an additional 3–4% of metastatic disease to sentinel lymph nodes can be detected (Kim et al., 2013). Since the use of the NCCN sentinel lymph node algorithm, the use of lymphadenectomy has decreased but the rate of detection of metastatic nodal involvement has not been affected.

While the lymph node drainage patterns of the endometrium are quite complex, the broad ligament is the most important drainage route, responsible for draining the mid and lower uterine corpus and the cervix, followed by the infundibulopelvic ligament, which drains the cornua to the renal vessels. The lymphatic drainage route should not be affected in a patient with a Müllerian anomaly. In the management of the patient described herein, the duplicated Müllerian system did not alter the method of lymph node mapping.

Most patients with endometrial carcinoma with uterine didelphys receive surgical management with total abdominal hysterectomy, bilateral salpingo-oophorectomy and peritoneal washings (Vanichtantikul et al., 2020). Our report is the first to document surgical management with sentinel lymph node mapping in a patient with endometrial carcinoma and uterine didelphys, despite the anatomic anomaly. Routine sentinel lymph node mapping can be successfully performed in this setting.

## 4 Consent

Written informed consent was obtained from the patient for publication of this case report and accompanying images. A copy of the written consent is available for review by the Editor-in-Chief of this journal on request.

## Funding

This study was funded in part through the NIH/NCI Support Grant P30 CA008748.

## Disclosures

**SC** is a consultant for AstraZeneca, outside the submitted work. **NRA** reports grants from Stryker/Novadaq, grants from Olympus, grants from GRAIL, outside the submitted work. Memorial Sloan Kettering Cancer Center (MSK) has financial interests relative to GRAIL. As a result of these interests, MSK could ultimately potentially benefit financially from the outcomes of this research. **ELJ** is a consultant for Intuitive Surgical Inc., an educational speaker for Covidien/Metronic, and reports personal fees from Covidien/Metronic, outside the submitted work.

## CRediT authorship contribution statement

**Dib Sassine:** Conceptualization, Data curation, Formal analysis, Writing - original draft, Writing - review & editing. **Sara Moufarrij:** Conceptualization, Data curation, Formal analysis, Writing - original draft, Writing - review & editing. **Anjelica Hodgson:** Data curation, Formal analysis, Writing - original draft, Writing - review & editing. **Sarah Ehmann:** Data curation, Formal analysis, Writing - review & editing. **Nadeem R. Abu-Rustum:** Formal analysis, Writing - review & editing. **Sarah Chiang:** Formal analysis, Writing - original draft, Writing - review & editing. **Elizabeth L. Jewell:** Conceptualization, Data curation, Formal analysis, Supervision, Writing - original draft, Writing - review & editing.

## Declaration of Competing Interest

The authors declare that they have no known competing financial interests or personal relationships that could have appeared to influence the work reported in this paper.
