# Supplementary material for: Case report: Sentinel lymph node mapping of endometrial carcinoma occurring in uterine didelphys
Source: Gynecol Oncol Rep. 2021 Apr 30;36:100769. doi: 10.1016/j.gore.2021.100769 (PMC8134959; doi:10.1016/j.gore.2021.100769)
Supplement: Supplementary data 1 [file mmc1.pdf]

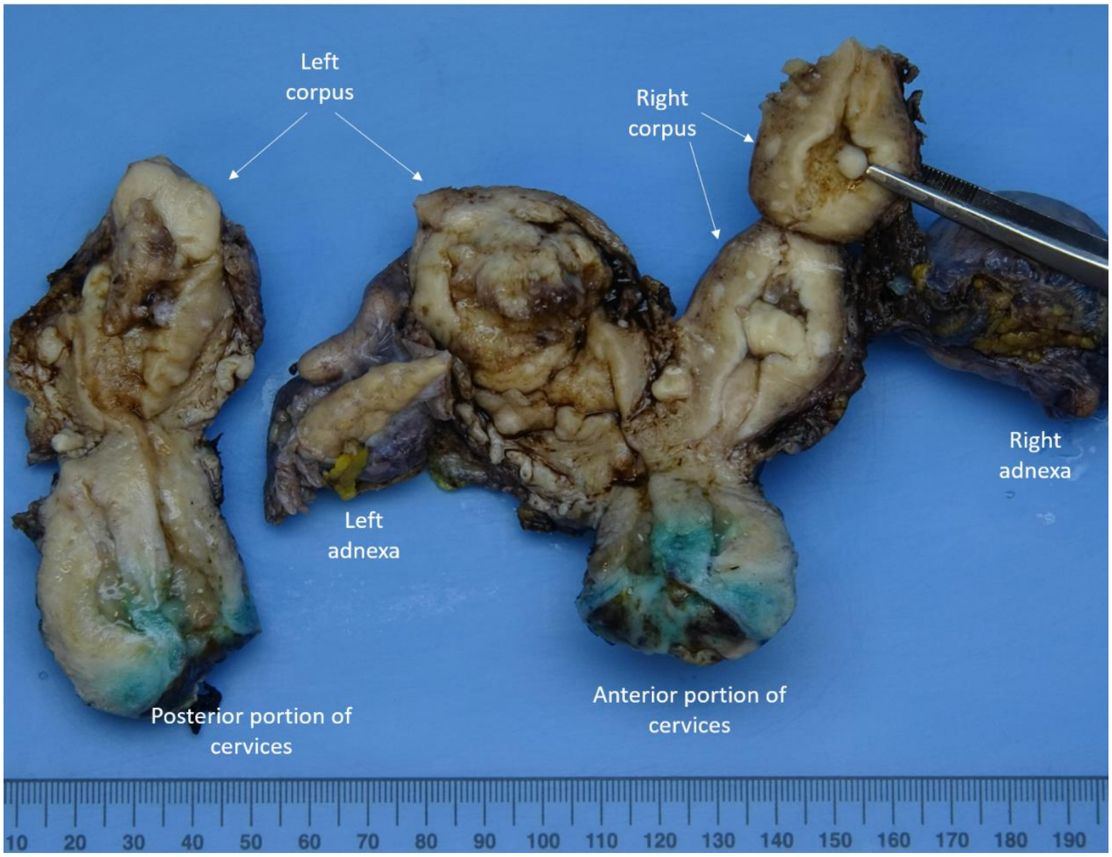
Left
corpus
Right
corpus
Right
adnexa
Left
adnexa
Anterior portion of
cervices
Posterior portion of
cervices
10 20 30 40 50 60 70 80 90 100 110 120 130 140 150 160 170 180 190

B

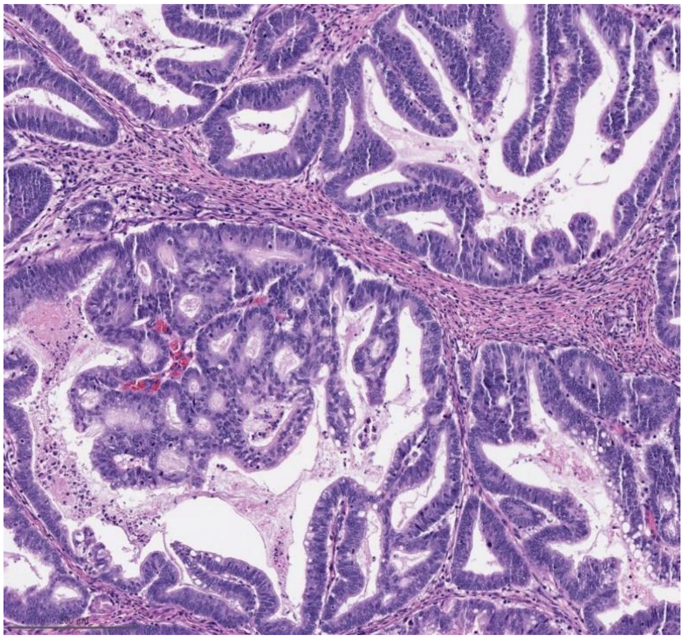

Supplementary Figure 1. Gross appearance of hysterectomy and bilateral salpingo-oophorectomy specimen. Note the endometrial-based tumor in the left corpus in addition to the rounded white polypoid lesion in the right corpus (adenomyoma). The right adnexa is enlarged due to a right ovarian simple cyst (A). Photomicrograph of endometrioid adenocarcinoma in the left-sided uterus. The tumor is composed of complex and cribriform glands lined by pseudostratified epithelium (magnification 100x; hematoxylin and eosin stain) (B).
